# New Literature

**DOI:** 10.1155/S1463924680000508

**Published:** 1980


### New Literature

### Water/wastewater analyses

A uv spectral instrument that measures total phenol in discrete water or wastewater samples and reports it as unsubstituted phenol (carbolic acid) is described in a bulletin from Fisher Scientific. The Fisher Phenol Analyser rerequires a 50 ml sample, two reagents, no sample dilution or pre-concentration over a 5 ppb to 20 ppm range, and distillation of only turbid or contaminated samples. Analysis of a single sample requires 3 minutes to 1 hour. Results are claimed to be less subject to variations in operator technique than conventional wet chemistry procedures.

*Fisher Scientific Co. 711 Forbes Ave, Pittsburgh, PA 15219, USA.*

### On-line carbon monitoring systems

A new range of on-line total carbon/total organic carbon monitoring systems, the Process/5000 series, has been developed by Ionics Inc. The range is now available in the UK from Techmation who will supply an 8-page booklet giving full details.

Four models are offered which monitor total carbon or total organic carbon on a continuously flowing sample by either high temperature combustion or uv oxidation reaction methods. The electronic design is computer-compatible. Analysers have response times from 6.5 minutes, and are recommended when the sample has a salt content of up to 30% or contains suspended solids up to 3,000 microns. Applications include the monitoring of drinking water and high purity process water, brines, high pressure boiler condensate and water used in semiconductor manufacture.

*Techmation Ltd, 58 Edgware Way, Edgware, Middx HA8 8JP, UK.*

### Lock-in amplifiers

The 1980 Lock-in Amplifier catalogue is now available from EG & G Princeton Applied Research Corporation. It features their complete line of lock-in amplifiers, preamplifiers, choppers, ratiometers and accessories. In addition to providing complete and detailed specifications including preamplifier noise figure contours, the catalogue includes lock-in amplifier comparison charts, application information, ordering information and a listing of their domestic and overseas field sales offices.

*EG & G Princeton Applied Research Corporation, Post Office Box 2565 Princeton, New Jersey 08540, USA*

### Quantitative determination of phenolic compounds in aqueous solutions

'The quantitative determination of phenolic compounds in aqueous solutions Part 1. Phenol equivalents of phenolic compounds using a continuous flow analysis technique' describes a continuous flow auto-analyser distillation method for determining the phenol equivalents of a number of phenolic compounds. The method used was one which is capable of handling soil extracts in 1 M NaOH, and was operated at a 150°C distillation temperature. The significance of the results is discussed. Recommendations are made for further investigations of (a) distillation at temperatures higher than 150°C, (b) the use of peak area rather than peak height to evaluate the results, and (c) the use of solvent extraction after the distillation stage in the continuous flow method.

*HMSO, 49 High Holborn, London WC1V 6HB, UK.*

### Japanese Journal of Clinical Laboratory Automation

### In volume 5, number 1, this journal will contain the following items:

**Panel discussion**
Some problems in the multiphasictest of few specimens by automated analyser

1. The evaluation of the Toshiba TBA-360, Shimazu CL-1, and Shimazu DIASPAT
S. Hayashi, *et al.* (2)
2. Application of the Abbott VP
N. Kobayashi, *et al.* (6)
3. Application of the Du Pont aca
E. Mizuno, *et al.* (8)
4. Application of the JCA-SI 6
H. Kushiro, *et al.* (13)
5. Application of the Hitachi 706 D
S. Sakano (16)

**Symposium**
Automated analysis of serum aspartate aminotransferase by the recommended method of the Japanese Society of Clinical Chemistry (JSCC)

I Remarks on the assay method for human serum GOT recommended by the JSCC
T. Horio, *et al.* (21)

II Determination of GOT by automated rate analyser using a reference method recommended by JSCC
J. Ohkawa (26)

III Some problems of automated instruments in the determination of enzyme activity – instrumental errors applying the recommended method to automated instruments
Y. Takahashi (30)

IV Applications for various instruments

1. Application of the Centrifichem 400 to the recommended method using a modified pipettor
Y. Katayama, *et al.* (34)
2. Application of centrifugal analysers
K. Yamamoto, *et al.* (38)
3. Application of the Hitachi 716
K. Morita, *et al.* (42)
4. Application of the Auto Analyser II
I. Furukawa, *et al.* (45)
5. Application of the Hitachi 706 D
H. Yamamichi, *et al.* (49)
6. Application of the JCA-SI 6
Y. Marui, *et al.* (53)

V Studies on the instrumentation of GOT determination using the reference method of the JSCC

1. Standard reference instrumentation relating to kinetic assay
K. Kuwa (57)
2. Determination of GOT using an improved TBA-360
Y. Murui, *et al.* (62)

VI The results and analysis of survey with GOT JSCC trial method using automated analyser
Z. Ogawa (67)

### Clinical analyser

A second generation discrete analyser is now available from Vitatron. The PA800 is a micro-computer based analyser on which both large batches and small numbers of samples can be run. A non-primed dispensing system means rapid test changeover and this, coupled with simplicity of operation, allows one operator to run all the analyses. It also allows the operator enough time on large batches of samples to perform other jobs round the laboratory. Additional features include full programming flexibility, small sample and reagent volumes and low running costs.

*MSE Scientific Instruments, Manor Royal, Crawley RH10 2QQ, UK.*

### Computing integrator

A computing integrator for gas and liquid chromatography has been introduced by Pye Unicam. The CDP1 computing integrator is designed around an 'easy key' control giving rapid familiarisation and simple operation. It is available with or without a digital display and has a built-in printer which outputs parameters, peak retention times and areas or heights, with baseline codes, followed by the calculated result.

A choice of manual, semi-automatic and automatic methods for peak detection ensures accurate area allocation throughout the analysis. A full self-integrity check with built-in signal generation to check correct integration, is also incorporated. Three built-in calculation methods are available, area % normalisation, normalisation with a scale factor, and an internal standard method.

*Pye Unicam Ltd, York St, Cambridge CB1 2PX, UK.*

### Calendar

**Editor's Note:**

***Organisers of conferences, seminars etc. should send details for inclusion in this calandar as soon as the relevant information is available and not later than three months before the event.***

**3rd International EPR Symposium**
August 11-14, Denver, U.S.A.
*Gareth R. Eaton, Department of Chemistry, University of Denver, Denver, Colorado, U.S.A.*

**Gordon Research Conference on Analytical Chemistry**

August 11-15, New Hampton.

*Gordon Research Conferences, Colby-Sawyer College, New London, NH 03257, U.S.A.*

**2nd Chemical Conference of the North American Continent**
August 24-29, San Francisco.
*A.P. Winstead, American Chemical Society 1155 16th St. NW, Washington, DC 20036, U.S.A.*

**7th European Congress on Electron Microscopy**

August 24-30, The Hague.

*Laboratory for Electron Microscopy, University of Leyden, Rijnsburgerweg 10, Leyden, The Netherlands.*

**Measurement and control of chemical hazards in the workplace environment**
August 25-28, San Francisco.
*Gangadhar Choudhary, National Institute for Occupational Safety and Health, DPSE, Mail Stop R3 4676 Columbia Parkway, Cincinnati, Ohio 45226, U.S.A.*

**12th Australian Spectroscopy Conference**

August 25-29, Sydney, Australia.

*Professor B.S. Orr, School of Chemistry, The University of New South Wales, PO Box 1, Kensington, NSW, Australia 2033.*

**11th International Congress of Clinical Chemistry — 4th European Congress of Clinical Chemistry**
August 30-September 5, Vienna
*Congress Secretariat, Interconvention, PO Box 35, A-1095 Vienna, Austria.*

**Immunoassay workshop**
August 31-September 5, Guildford, U.K.
*Courses Secretary, Department of Biochemistry, University of Surrey, Stag Hill, Guildford GU2 5XH, U.K.*

**Annual meeting of the British Association for the Advancement of Science**

September 1-5, Salford.

*Miss J.H. Dring, BAAS, 23 Saville Row, London W1, U.K.*

**Laboratory methods and techniques**
September 2-4, Portsmouth.
*J.M. Potts, Room 306, Chemistry Dept, St Michael's Building, Portsmouth Polytechnic, White Swan Road, Portsmouth PO1 2DT, U.K.*

**12th Annual Conference of the European Group for Atomic Spectroscopy**
September 2-5, Pisa.
*A. Gozzini, Instituto di Fisica, Universita di Pisa, Piazza Torricelli 2, 56100 Pisa, Italy.*

**International solvent extraction conference**
September 6-12, Liege, Belgium.
*Conference Secretary, ISEC 80, Dept of Chemistry, University of Liege, Sart Tilman, B-4000 Liege, Belgium.*

**3rd World Filtration Congress**

September 13-17, Philadelphia.

*Filtration Society, Shippensburg, Philadelphia, U.S.A.*

**Modern techniques in centrifugation**

September 14-19, Colchester.

*Liaison Officer, University of Essex, Wivenhow Park, Colchester CO4 3SQ, U.K.*

**6th International Symposium on Advances and Applications of Chromatography in Industry**
September 16-19, Bratislava, Czechoslovakia.
*Jan Remen, Analytical Section CS VTS, pri np Slovnaft, 82300, Bratislava, Czechoslovakia.*

**Clinical laboratory automation**
September 19-20, Tokyo.
*Japanese Society of Clinical Laboratory Automation, 9-3 Nakamarucho, Itabashi-ku, Tokyo 173, Japan.*

**High performance liquid chromatography course**
September 22-26, Loughborough.
*Miss J.M. Brown, Department of Chemistry, Loughborough University of Technology, Loughborough, Leics. LE11 3TU, U.K.*

**Trace and ultratrace analysis**
September 23-25, Cardiff.
*The Secretary, Analytical Division, Chemical Society, Burlington House, London W1V 0BN, U.K.*

**7th Annual meeting of the Federation of Analytical Chemistry and Spectroscopy Societies**
September 28-October 3, Philadelphia.
*S.W. Fleming, Engineering Physics Laboratory, Du Pont Experimental Station, Wilmington, Del 19898, U.S.A.*

**Analysis 80 — The installation and management of micro and mini computers in the laboratory**
September 29-30, London.
*Scientific Symposia Ltd, 33-35 Bowling Green Lane, London EC1R 0DA, U.K.*

**ASTM E-19 Meeting on the Theory and Practice of Chromatography**
October 1-3, Pennsylvania State University.
*Clayton O. Ruud, 159 Materials Research Laboratory, Pennsylvania State University, University Park, Pa 16892, U.S.A.*

